# Draft Genome Sequences of Two *Aspergillus fumigatus* Strains, Isolated from the International Space Station

**DOI:** 10.1128/genomeA.00553-16

**Published:** 2016-07-14

**Authors:** Nitin Kumar Singh, Adriana Blachowicz, Aleksandra Checinska, Clay Wang, Kasthuri Venkateswaran

**Affiliations:** a Biotechnology and Planetary Protection Group, Jet Propulsion Laboratory, California Institute of Technology, Pasadena, California, USA; b Department of Pharmacology and Pharmaceutical Sciences, University of Southern California, Los Angeles, California, USA; c Dornsife College of Letters, Arts and Sciences, University of Southern California, Los Angeles, California, USA

## Abstract

Draft genome sequences of *Aspergillus fumigatus* strains (ISSFT-021 and IF1SW-F4), opportunistic pathogens isolated from the International Space Station (ISS), were assembled to facilitate investigations of the nature of the virulence characteristics of the ISS strains to other clinical strains isolated on Earth.

## GENOME ANNOUNCEMENT

Fungi are a source of natural products that are useful to humankind. A variety of bioactive compounds produced by fungi are used in pharmaceutical and food industries, agriculture, and beyond. However, some fungi are plant, animal, and human pathogens. Fungal pathogens could cause serious health complications in immunocompromised populations. Pathogenic fungi can produce a range of secondary metabolites (SMs) that influence their virulence (melanins, siderophores, species-specific toxins) and immunologic potential.

*Aspergillus fumigatus* is a saprophytic, filamentous fungus that is ubiquitous outdoors (soil, decaying vegetation) and indoors (hospitals, simulated closed habitats, etc.). *Aspergillus fumigatus* can adapt to various environmental conditions and form airborne conidia that are the inoculum for a variety of diseases (e.g., noninvasive and invasive pulmonary infections, allergic bronchopulmonary aspergillosis, etc.) in immunocompromised hosts.

In on-going microbial observatory experiments on the International Space Station (ISS), molecular phylogeny and radiation resistance of several fungal isolates were characterized. Two *Aspergillus fumigatus* strains, ISSFT-021 and IF1SW-F4, were isolated from a HEPA filter (1) and cupola wall (unpublished) of the ISS. Because *A. fumigatus* is an opportunistic pathogen causing pathologies ranging from allergic asthma to invasive aspergillosis, we assessed several pathogenic characteristics of the ISS isolates in comparison to two experimentally established clinical isolates, Af293 (2) and CEA10 (3). Virulence assessment in a larval zebrafish model of invasive aspergillosis revealed both ISSFT-021 and IF1SW-F4 as significantly more lethal compared to both clinical isolates (Af293 and CEA10) (4). In addition, the ISS strains ISSFT-021 and IF1SW-F4 exhibited significantly greater resistance to UV254 doses when compared to clinical isolates (4, 5). The genomics of these ISS isolates might reveal the molecular mechanisms of the increased virulence. Subsequently, if the enhanced virulence is attributed to the microgravity, NASA potentially should consider developing countermeasures to protect the health of astronauts, whose immune systems are reported to be compromised under microgravity (6).

In this study, we determined the draft genome sequences of *A. fumigatus* strains ISSFT-021 and IF1SW-F4. Whole-genome shotgun sequencing was performed on an Illumina HiSeq2500 instrument with a paired-end module. A total of 27,987,752 and 10,752,032 paired-end reads of 101-nucleotides were collected for ISSFT-021 and IF1SW-F4. The NGS QC Toolkit v2.3 (7) was used to filter the data for high-quality (HQ) vector- and adaptor-free reads for genome assembly (cutoff read length for HQ, 80%; cutoff quality score, 20). A total of 25,515,334 (~230× coverage) for ISSFT-021 and 8,466,430 (~75× coverage) for IF1SW-F4 high-quality vector-filtered reads were used for assembly with MaSuRCA (8) genome assembler (k-mer size = 70). The final assembly of the strain ISSFT-021 contains 301 scaffolds with a total size of 28,526,023 bp and an *N*_50_ contig length of 275.468 kb; the largest contigs assembled measures 813.103 kb and the G+C% was 49.41. Similarly, the final assembly of the IF1SW-F4 strain contains 208 scaffolds with a total size of 28,240,437 bp and an *N*_50_ contig length of 367.421 kb; the largest contig assembled measures 900.278 kb and the G+C content was 49.45%.

### Nucleotide sequence accession numbers.

The whole-genome sequences of both the strains were deposited at DDBL/EMBL/GenBank under the accession numbers LWRT00000000 and LWRU00000000. The versions described in this paper are LWRT01000000 and LWRU01000000. These *A. fumigatus* strains were also deposited in the USDA Agricultural Research Station and DSMZ public culture collections.

## References

[1] Checinska A, Probst AJ, Vaishampayan P, White JR, Kumar D, Stepanov VG, Fox GE, Nilsson HR, Pierson DL, Perry J, Venkateswaran K 2015 Microbiomes of the dust particles collected from the International Space Station and Spacecraft Assembly Facilities. Microbiome 3:50. doi:10.1186/s40168-015-0116-3. 26502721 PMC4624184

[2] Nierman WC, Pain A, Anderson MJ, Wortman JR, Kim HS, Arroyo J, Berriman M, Abe K, Archer DB, Bermejo C, Bennett J, Bowyer P, Chen D, Collins M, Coulsen R, Davies R, Dyer PS, Farman M, Fedorova N, Fedorova N, Feldblyum TV, Fischer R, Fosker N, Fraser A, Garcia JL, Garcia MJ, Goble A, Goldman GH, Gomi K, Griffith-Jones S, Gwilliam R, Haas B, Haas H, Harris D, Horiuchi H, Huang J, Humphray S, Jimenez J, Keller N, Khouri H, Kitamoto K, Kobayashi T, Konzack S, Kulkarni R, Kumagai T, Lafon A, Latge JP, Li W, Lord A, Lu C, Majoros WH, May GS, Miller BL, Mohamoud Y, Molina M, Monod M, Mouyna I, Mulligan S, Murphy L, O’Neil S, Paulsen I, Penalva MA, Pertea M, Price C, Pritchard BL, Quail MA, Rabbinowitsch E, Rawlins N, Rajandream MA, Reichard U, Renauld H, Robson GD, Rodriguez de Cordoba S, Rodriguez-Pena JM, Ronning CM, Rutter S, Salzberg SL, Sanchez M, Sanchez-Ferrero JC, Saunders D, Seeger K, Squares R, Squares S, Takeuchi M, Tekaia F, Turner G, Vazquez de Aldana CR, Weidman J, White O, Woodward J, Yu JH, Fraser C, Galagan JE, Asai K, Machida M, Hall N, Barrell B, Denning DW 2005 Genomic sequence of the pathogenic and allergenic filamentous fungus *Aspergillus fumigatus*. Nature 438:1151–1156. doi:10.1038/nature04332. 16372009

[3] Rizzetto L, Giovannini G, Bromley M, Bowyer P, Romani L, Cavalieri D 2013 Strain dependent variation of immune responses to *A. fumigatus*: definition of pathogenic species. PLoS One 8:e56651. doi:10.1371/journal.pone.0056651. 23441211 PMC3575482

[4] Knox BP, Blachowicz A, Romsdahl J, Palmer JM, Huttenlocher A, Wang CCC, Keller NP, Venkateswaran K 2016 7th Advances Against Aspergillosis Conference, Manchester, United Kingdom, 3 3 to 5, 2016.

[5] Blachowicz A, Knox BP, Romsdah LJ, Palmer JM, Huttenlocher A, Wang CCC, Keller NP, Venkateswaran K 2016 Characterization of *Aspergillus fumigatus* isolated from air and surfaces of the International Space Station, 13th European Conference on Fungal Genetics, Paris, France, 4 3 to 6, 2016.

[6] Crucian BE, Zwart SR, Mehta S, Uchakin P, Quiriarte HD, Pierson D, Sams CF, Smith SM 2014 Plasma cytokine concentrations indicate that in vivo hormonal regulation of immunity is altered during long-duration spaceflight. J Interferon Cytokine Res 34:778–786. doi:10.1089/jir.2013.0129. 24702175 PMC4186776

[7] Patel RK, Jain M 2012 NGS QC toolkit: a toolkit for quality control of next generation sequencing data. PLoS One 7:e30619. doi:10.1371/journal.pone.0030619. 22312429 PMC3270013

[8] Zimin AV, Marçais G, Puiu D, Roberts M, Salzberg SL, Yorke JA 2013 The MaSuRCA genome assembler. Bioinformatics 29:2669–2677. doi:10.1093/bioinformatics/btt476. 23990416 PMC3799473

